# In vitro selection of resistance in *Escherichia coli *and *Klebsiella *spp. at *in vivo *fluoroquinolone concentrations

**DOI:** 10.1186/1471-2180-10-119

**Published:** 2010-04-21

**Authors:** Lorenzo Drago, Lucia Nicola, Roberto Mattina, Elena De Vecchi

**Affiliations:** 1 Laboratory of Microbiology, Dept Preclinical Sciences LITA Vialba, University of Milan, Via GB Grassi 74, Milan 20157, Italy and Laboratory of Clinical Chemistry IRCCS Galeazzi, Via R. Galeazzi 4, Milan 20161, Italy; 2 Department of Public Health, Microbiology and Virology, University of Milan, Via Pascal, Milan 20166, Italy

## Abstract

**Background:**

Fluoroquinolones are potent antimicrobial agents used for the treatment of a wide variety of community- and nosocomial- infections. However, resistance to fluoroquinolones in Enterobacteriaceae is increasingly reported. Studies assessing the ability of fluoroquinolones to select for resistance have often used antimicrobial concentrations quite different from those actually acquired at the site of infection. The present study compared the ability to select for resistance of levofloxacin, ciprofloxacin and prulifloxacin at concentrations observed *in vivo *in twenty strains of *Escherichia coli *and *Klebsiella *spp. isolated from patients with respiratory and urinary infections. The frequencies of spontaneous single-step mutations at plasma peak and trough antibiotic concentrations were calculated. Multi-step selection of resistance was evaluated by performing 10 serial cultures on agar plates containing a linear gradient from trough to peak antimicrobial concentrations, followed by 10 subcultures on antibiotic-free agar. *E. coli *resistant strains selected after multi-step selection were characterized for DNA mutations by sequencing *gyrA*, *gyrB*, *parC *and *parE *genes.

**Results:**

Frequencies of mutations for levofloxacin and ciprofloxacin were less than 10^-11 ^at peak concentration, while for prulifloxacin they ranged from <10^-11 ^to 10^-5^. The lowest number of resistant mutants after multistep selection was selected by levofloxacin followed by ciprofloxacin and prulifloxacin. Both ciprofloxacin- and prulifloxacin-resistant mutants presented mutations in *gyrA *and *parC*, while levofloxacin resistance was found associated only to mutations in *gyrA*.

**Conclusions:**

Among the tested fluoroquinolones, levofloxacin was the most capable of limiting the occurrence of resistance.

## Background

*Escherichia coli *is worldwide the most frequent pathogen isolated from uncomplicated urinary tract infections (UTI) (70 - 95%) and, in bacteremia of nosocomial or community origin, it represents about the 15.5% and 42.1% of aetiologies, respectively [1]. Also *Klebsiella *spp., especially *Klebsiella pneumoniae*, are involved in uncomplicated UTI for 5% and represent 4.1% of bacteremias, the mortality of nosocomial infections being more than twice that of community-acquired infection [1,2].

Fluoroquinolones (FQ) are potent antimicrobial agents used for the treatment of a wide variety of community- and nosocomial- infections. However, increasing resistance to FQ in *E. coli *isolated from community acquired UTI has been recently reported, with up to 29% of women harbouring FQ resistant *E. coli*, although FQ resistance rates varied significantly according to sex, age, type of urinary infection and geographic region [3-6]. Moreover, infections due to extended-spectrum beta-lactamases (ESBL) - producing Enterobacteriaceae are an emerging problem in the community since an high proportion of these microorganisms have been isolated from urine samples of women with uncomplicated UTI [7].

Ciprofloxacin use and ESBL production have been shown to be significantly correlated in a study on *K. pneumoniae *[8]. ESBL-producing strains have been shown to be significantly more frequent among ciprofloxacin-resistant *E. coli *than among ciprofloxacin-susceptible *E. coli *strains [9]. Moreover, prior use of FQs, an indwelling urinary catheter, and an invasive procedure within 72 hr prior to bacteremia have been identified as independent risk factors for ciprofloxacin resistance in bloodstream infections due to ESBL *E. coli *and *Klebsiella *spp. [2,10-12].

Several studies have assessed the ability of FQs to select for resistance by subculturing bacteria at concentrations close to MICs. However, the antimicrobial concentrations used in these studies were quite different from those actually acquired at the site of infection [13-16]. For these reasons, we have recently modified the methodologies used to assess *in vitro *the selection for resistance by testing antimicrobial concentrations reported to occur *in vivo *[17]. The aim of the present study was to compare the ability of levofloxacin, ciprofloxacin and prulifloxacin to *in vitro *select for resistance in *E. coli *and *Klebsiella *spp. clinical isolates at peak (Cmax) and trough (Cmin) plasma concentrations.

## Results

### Susceptibility to fluoroquinolones

Basal MICs of *E. coli *strains ranged from 0.016 mg/L to 1 mg/L, from 0.004 mg/L to 0.5 mg/L and from 0.016 mg/L to 0.125 mg/L for levofloxacin, ciprofloxacin and prulifloxacin, respectively. MICs of *Klebsiella *spp. ranged between 0.03 mg/L and 1 mg/L, 0.016 mg/L and 0.5 mg/L, and 0.03 and 0.25 mg/L for levofloxacin, ciprofloxacin and prulifloxacin, respectively.

### Frequency of mutation

Levofloxacin, 500 and 750 mg, and ciprofloxacin 500 mg limited bacterial growth with median frequencies of mutations below 10^-11 ^at plasma Cmax. Median frequencies of mutations for prulifloxacin were generally higher than comparators ranging from 10^-7 ^to 10^-8 ^and from 10^-8 ^to 10^-9 ^at plasma Cmax in *E. coli *and *Klebsiella *spp., respectively (Table 1). Table 2 shows MIC values of the strains that were able to grow in the presence of the above mentioned concentrations of all tested antimicrobials. While no strain was able to grow at Cmax for levofloxacin and ciprofloxacin, 3 and 5 strains grew at prulifloxacin Cmax. These strains showed increments in MICs from 32 to 128 times for *E. coli *and from 32 to 128 times for *Klebsiella *spp. with respect to the basal values. Since in some instances, Cmin for all the study drugs, except for levofloxacin at 750 mg dosage, were below MIC values, some strains were able to diffusely grow on the agar plate. For these strains, in order to detect any change in bacterial susceptibility, MICs were evaluated for randomly sampled colonies (Table 2).

**Table 1:** Frequency of mutation at plasma antimicrobial concentrations in *E. coli *and *Klebsiella spp*.

Drug	Frequency of mutation			
	
	*E. coli *(n = 20)		*Klebsiella spp*. (n = 20)	
	Cmax	Cmin *	Cmax	Cmin*
**LVX 500 mg**				
**Range**	<10^-11^	< 10^-11 ^- 1.0 × 10^-7^	<10^-11^	<10^-11 ^- 7.4 × 10^-5^
**median**	<10^-11^	2.0 × 10^-11^	<10^-11^	7.9 × 10^-8^
**LVX 750 mg**				
**Range**	<10^-11^	<10^-11 ^- 2.7 × 10^-5^	<10^-11^	<10^-11 ^- 7.7 × 10^-6^
**median**	<10^-11^	<10^-11^	<10^-11^	2.2 × 10^-8^
**CIP 500 mg**				
**Range**	<10^-11^	<10^-11 ^- 6.3 × 10^-6^	<10^-11^	3.2 × 10^-8 ^- 8.5 × 10^-5^
**median**	<10^-11^	<10^-11^	<10^-11^	1.5 × 10^-7^
**PRU 600 mg**				
**Range**	<10^-11 ^- 2.4 × 10^-6^	< 10^-11 ^- 4.1 × 10^-6^	<10^-11 ^- 1.7 × 10^-5^	6.3 × 10^-9^- 2.2 × 10^-5^
**median**	4.3 × 10^-8^	2.4 × 10^-7^	6.7 × 10^-9^	7.1 × 10^-7^

LVX: Levofloxacin; CIP: Ciprofloxacin; PRU: Prulifloxacin; Cmax: peak plasma concentration; Cmin: trough plasma concentration

* Frequency of mutations was calculated only for strains with MIC < Cmin.

**Table 2:** Fluoroquinolone activity on strains grown after single step selection in *E. coli *and *Klebsiella spp*. at plasma concentrations

Drug	MIC range (mg/L)/number of strains grown			
	
	*E. coli *(n = 20)		*Klebsiella spp*. (n = 20)	
	
	Cmax	Cmin*	Cmax	Cmin*
**LVX **500 mg	-/0	1/1	-/0	0.5 - 4/16
**LVX **750 mg	-/0	1 - 4/2	-/0	1 - 8/14
**CIP **500 mg	-/0	0.25 - 0.5/4	-/0	0.125 - 4/20
**PRU **600 mg	2 - 4/3	0.25 - 2/5	4 - 8/5	0.06 - 1/20

LVX: Levofloxacin; CIP: Ciprofloxacin; PRU: Prulifloxacin; Cmax: peak plasma concentration; Cmin: trough plasma concentration

* MICs were evaluated for all the tested strains

### Multi-step selection of resistant bacteria

Table 3 shows the total number of strains grown after multi-step selection and MIC values after 1, 5 and 10 passages on antibiotic-gradient plates and after the subsequent 10 passages on antibiotic-free medium. After multi-step selection, a general increment in MICs was observed for all microrganisms with all tested antibiotics; no selection of resistance was observed with levofloxacin at 750 mg in *E. coli *and no selection of resistance was observed with levofloxacin (both doses) in *Klebsiella *spp.

**Table 3:** MIC values after multi-step selection of resistance in *E. coli and Klesiella spp*. at plasma concentration of fluoroquinolones

Drug	MIC (mg/L): median (range)					
	
	Nr of strains	Pre-sel	I STEP	V STEP	X STEP	X STEP free
***E. coli (n = 20)***						
**LVX** **500 mg**	7	0.5 (0.5 - 1)	2 (0.5-4)	4 (1 - 8)	8 (2 - 8)	4 (1 - 8)
**LVX** **750 mg**	0	0.016 - 1	n.d.	n.d.	n.d.	n.d.
**CIP** **500 mg**	8	0.25 (0.125 - 0.5)	0.5 (0.125 - 1)	2 (2 - 4)	8 (4 - 16)	4 (1 - 8)
**PRU** **600 mg**	12	0.064 (0.016 - 0.125)	1 (0.5 - 4)	2 (2 - 4)	4 (2 - 8)	4 (2 - 8)
***Klebsiella spp. (n = 20)***						
**LVX** **500 mg**	0	0.03 - 1	n.d	n.d	n.d	n.d
**LVX** **750 mg**	0	0.03 - 1	n.d	n.d	n.d	n.d
**CIP** **500 mg**	11	0.06 (0.03 - 0.5)	0.5 (0.5 - 1)	2 (1 - 8)	8 (4 - 16)	4 (1 - 4)
**PRU** **600 mg**	16	0.06 (0.03 - 0.25)	0.5 (0.06 - 1)	2 (0.25 - 16)	4 (0.5 - 32)	4 (0.25 - 16)

LVX: Levofloxacin; CIP: Ciprofloxacin; PRU: Prulifloxacin; Pre-sel: MICs before starting multi-step selection of resistance; I Step: MICs after the first passage on antibiotic gradient agar plates; V Step: MICs after the fifth passage on antibiotic gradient agar plates; X Step: MICs after the last passage on antibiotic gradient agar plates; X step free: MICs after ten subcultures on antibiotic free agar plates.

**Figure 1:**
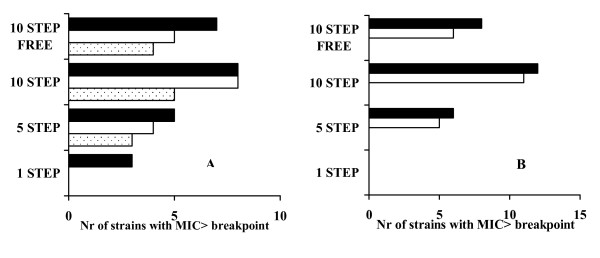
**Multi-step selection of resistance in *E. coli *(A) and *Klebsiella *spp. (B) at plasma concentration of fluoroquinolones**. 1, 5, 10 step: number of passages on antibiotic gradient agar plates. 10 step free: passages on antibiotic free agar plates. Black bars: prulifloxacin; White bars: ciprofloxacin; Dotted bars: levofloxacin.

After 10 passages on antibiotic gradient plates and 10 subcultures in antibiotic-free medium, the highest number of strains with MIC higher than the resistance breakpoint was found for ciprofloxacin and prulifloxacin both in *E. coli *(5 and 7 strains, respectively) and *Klebsiella *spp. (6 and 8 strains, respectively). Only 4 strains with MIC higher than resistance breakpoint were found with levofloxacin at 500 mg in *E. coli *and *Klebsiella *spp., whereas no resistant strains selected with the 750 mg either in *E. coli *or in *Klebsiella *spp. (Figure 1).

### Characterization of acquired resistance

Strains of *E. coli *that were selected by the multi-step assay and were able to maintain their resistance after 10 passages in antibiotic-free medium, were evaluated for acquired resistance.

Among 16 resistant mutants, alterations in both *gyrA *and *parC *were found in 12 mutants for ciprofloxacin (n = 5) and prulifloxacin (n = 7), while only alterations in *gyrA *were found for levofloxacin. As reported in table 4, the 4 strains resistant to levofloxacin showed changes in Ser83Leu and Asp87Asn; while in ciprofloxacin- and prulifloxacin-resistant mutants, the mutations identified were Ser83Leu in GyrA and Ser80Ile in ParC. The same mutations were not found in the respective parent strains.

**Table 4:** Amino acid changes encoded by mutations in *gyrA, gyrB, parC*, and *parE *in *E. coli*

	Replacement in QRDR			
	
Drug	GyrA	GyrB	ParC	ParE
LVX (n = 4)	Ser83Leu (4) Asp87Asn (4)	-	-	-
CIP (n = 5)	Ser83Leu (5)	-	Ser80Ile (5)	-
PRU (n = 7)	Ser83Leu (7)	-	Ser80Ile (7)	-

## Discussion

Wild-type *E. coli *and *K. pneumoniae *clinical isolates are susceptible to quinolones, but resistance to these agents in Gram-negative bacteria has increased in recent years, probably caused by excessive and inappropriate use of these drugs [18]. Particularly, due to under-dosing and mono-therapy against moderately susceptible pathogens, FQ resistance has developed among common pathogens, like *E. coli *and *Klebsiella *spp., mainly conferred by ESBLs and AmpC enzymes [19]. ESBL production has been reported to be two times more common in infected patients who received ciprofloxacin than in those who did not (15% vs 7.4%) [8].

In a study performed over 5 years in Croatia on changes in susceptibility of *E. coli *from UTI, Moeal et al have shown a statistically significant change in antimicrobial resistance over that period only for ciprofloxacin [20]. This has been hypothesized to be related to the inappropriate use of quinolones for humans as well as in veterinary medicine [21]. Prolonged use (> 20 days) of low dose (250 mg twice a day) of the more potent fluoroquinolones such as ciprofloxacin or levofloxacin, has been shown to be the most significant risk factor for acquisition of resistance [22,23]. Strategies to counteract bacterial resistances include use of the appropriate dosages of these molecules for the correct indication and/or use of synergistic combinations, particularly in the more complicated infections.

Results of this study indicate that levofloxacin presents the lowest frequencies of mutations at plasma Cmax (<10^-11^) and a lower propensity than prulifloxacin or ciprofloxacin to select *in vitro *for resistance. In regard to genetic characterization of resistance, only alterations in *gyrA *were found for levofloxacin, however, alterations in *gyrA *and *parC *were found for ciprofloxacin and prulifloxacin. Point mutations within DNA gyrase are known to cause a reduction in the affinity of the enzyme for FQs, decreasing the susceptibility of bacteria to these molecules. Topoisomerase IV is the second target for FQ in the absence of susceptible gyrase. Therefore, multiple mutations in *gyrA *and/or *parC *are required for high level FQ resistance in *E. coli *[23,24]. In our study, both ciprofloxacin and prulifloxacin resistant mutants presented mutations in *gyrA *and *parC*, while levofloxacin resistance was found associated only with mutations in *gyrA*. These results seem to indicate that levofloxacin resistance at a concentration observed during treatment might develop more slowly and might be lower than resistance to the other FQs tested in the present study.

However, this study did not evaluated other mechanisms other than the target enzyme that might be involved in the observed resistant strains, including decreased intracellular drug accumulation as a result of alterations in the outer membrane proteins of the wall cell, or active efflux of the drug mediated by a number of efflux pumps.

As far as FQ resistance in *Klebsiella spp*. is concerned, plasmid-mediated quinolone resistance mechanisms associated with the *qnr *gene and the *aac(6')-Ib-cr *gene in ESBL producing strains have been described [25,26]. The first encodes target protection proteins of the pent peptide repeat family and seems to be associated with low level quinolone resistance, while the *aac(6')-Ib-cr *gene encodes a variant of the common aminoglycoside acetyltransferase which is able to reduce the activity of some FQ, thus enhancing the selection of chromosomal mutations [25]. Although in the present study the presence of plasmid-mediated resistance was not investigated, it can not be excluded that these genes might be involved in selection of resistance observed after serial exposure to fluoroquinolones.

In a previous study, we have shown that combinations of a fluoroquinolone with a beta-lactam may both provide improved antimicrobial activity and limit the occurrence of resistance in ESBL-producing *E. coli *clinical isolates [27]. Therefore, the use of combination therapy could be an attractive strategy to limit occurrence of resistance.

## Conclusions

In conclusion, among the tested fluoroquinolones, levofloxacin was the most able to limit occurrence of resistance *in vitro*. However, in order to limit the occurrence of resistance, appropriate dosages of fluoroquinolones should be respected in the therapy of infections caused by Enterobacteriaceae, as well as use of synergistic combinations in the most complicated infections.

## Methods

### Strains

Twenty clinical isolates of *E. coli *and *Klebsiella *spp., collected from patients presenting with community infections in 2005 at L. Sacco Hospital, Milan, were included into the study. Susceptibility to the drugs under evaluation was considered as a pre-requisite for the study. One isolate per patient was used in order to avoid inclusion of the same strain. All isolates were stored at -80°C in brain-heart infusion broth containing 10% (w/v) glycerol until use.

### Antibiotics

Levofloxacin (sanofi-aventis, S.p.A. Milan, Italy); ciprofloxacin (Bayer Italia, S.p.A., Milan, Italy), and prulifloxacin (Aziende Chimiche Riunite Angelini Francesco ACRAF S.p.A, S. Palomba-Pomezia, Italy) were used to prepare stock solutions at concentrations of 5120 mg/L. Plasma maximum and minimum concentrations (Cmax, Cmin) of each antimicrobial studied were chosen from those obtained at steady state in previously published studies after oral administration [28-31]. Thus, the Cmax were as following: levofloxacin 500 mg (5.29 mg/L); levofloxacin 750 mg (11.98 mg/L); ciprofloxacin 500 mg (2.11 mg/L); prulifloxacin 600 mg (2 mg/L) [28-31]. The tested plasma Cmin were respectively: 0.60 mg/L for levofloxacin 500 mg; 1.69 mg/L for levofloxacin 750 mg; 0.08 mg/L for ciprofloxacin 500 mg; 0.10 mg/L for prulifloxacin 600 mg [28-31].

### Determination of MIC

Antibiotic susceptibilities to the study drugs were determined by the microdilution broth assay in accordance with CLSI approved standards [32]. Since no CLSI breakpoints for prulifloxacin against *E. coli *and *Klebsiella *spp. were available, reduced susceptibility to this agent was defined as a MIC ≥ 4 mg/L [32]. Resistance to levofloxacin and ciprofloxacin was defined by MIC values ≥ 8 and 4 mg/L, respectively [33].

### Frequency of mutation

Colonies from an overnight culture in Mueller Hinton agar were resuspended in brain heart infusion (BHI) broth at a load of about 10^10 ^CFU/mL. An aliquot of 100 μL from the bacterial suspension was spread onto Mueller Hinton agar plates containing antibiotics at plasma Cmax and Cmin, as reported above. After incubation for 72 h, the frequency of mutation was calculated from the ratio between colonies grown on antibiotic-containing plates and the initial inoculum, determined by plating 100 μL of bacterial suspension, after proper dilution, onto Mueller Hinton agar plates. Five colonies from each antibiotic containing plate were randomly selected and their MIC for the corresponding antibiotic was determined as described above. When MIC was higher than the tested concentration, as occurred for Cmin for some strains, so that colony counts was not possible because of extensive growth on plate surface, frequency of mutation was not calculated, but the MIC was equally determined.

### Multi-step selection of resistant bacteria

The ability to select for antibiotic resistance was evaluated by performing serial subcultures on Mueller Hinton agar plates, containing a gradient ranging from Cmax to Cmin. Gradients were prepared in Petri dishes, which were poured with two layers of agar, as described elsewhere [34]. The bottom layer consisted of Mueller Hinton agar containing the antibiotic at Cmin, which was allowed to harden with the plate slanted sufficiently to cover the entire bottom. The top layer, added to the dish in the normal position, contained antibiotics at Cmax.

An inoculum of 10^10 ^CFU/mL of each strain was homogenously spread onto each plate and incubated for 48 hrs at 37°C. After incubation, colonies grown at the highest drug concentration were sampled, checked for purity, and re-plated on a new antibiotic-containing agar plates. A total of 10 consecutive passages on antibiotic containing plates were followed by 10 passages on antibiotic-free plates in order to evaluate stability of acquired resistance. MIC values were determined after 1, 5 and 10 passages on antibiotic containing plates and after 5 and 10 passages in antibiotic free medium in order to evaluate stability of acquired resistance. Acquisition of resistance was defined as a MIC value higher than resistance breakpoint.

### Characterization of acquired resistance

To determine whether *E. coli *mutants that had acquired stable resistance to quinolones had alterations in topoisomerase IV or DNA gyrase, *parC, parE, gyrA*, and *gyrB *were amplified by PCR and sequenced as described previously [35].

Amplification products were purified with the QIAquick PCR purification kit (Qiagen Inc., Milan Italy) using the manufacturer's instructions. Sequencing was performed on an ABI PRISM 310 genetic analyzer (Applied Biosystems, Monza, Italy).

Only mutations known to be associated with resistance to fluoroquinolones were considered (Ser83, Asp87 and Ala93 in GyrA, Ser80 and Glu84 in ParC) [36].

## Competing interests

This work was supported by an unrestricted grant from sanofi-aventis. L. Drago has acted as a speaker for sanofi-aventis.

## Authors' contributions

LD participated in designing the study, data analysis and in the writing of the paper. LN performed all experiments and participated in data collection and analysis. RM participated in writing of the paper. EDV participated in designing the study, data analysis and in the writing of the paper. All authors read and approved the final manuscript.
